# Prenatal diagnosis and postnatal management of congenital mesoblastic nephroma: A case report and literature review

**DOI:** 10.3389/fped.2022.1040304

**Published:** 2022-11-21

**Authors:** Xiaoxiao Zhang, Huijing Zhang, Shuang Wang, Yangxu Gao, Li Liang, Huixia Yang

**Affiliations:** ^1^ Department of Obstetrics and Gynecology, Peking University First Hospital, Beijing, China; ^2^ Department of Pediatric Surgery, Peking University First Hospital, Beijing, China; ^3^ Department of Pathology, Peking University First Hospital, Beijing, China

**Keywords:** congenital mesoblastic nephroma (CMN), prenatal ultrasound, solid renal mass, nephrorectomy, CLASSIC

## Abstract

**Background:**

Congenital mesoblastic nephroma (CMN) is a rare renal tumour in children, the most common kidney tumour in the neonatal period. It can be divided into three types, classical, cellular and mixed.

**Case presentation:**

A 31-year-old Chinese woman had no apparent foetal abnormality in regular prenatal care during the first and second trimesters. At 33 weeks of gestation, a solid mass in the right kidney was noted with echoes similar to liver and hypervascularity. It grew larger during late pregnancy. The infant was transferred to have a radical nephrectomy on the 9th day after birth. The postoperative histopathological result indicated classical CMN.

**Conclusion:**

CMN could be detected prenatally, mainly during late pregnancy. The postnatal outcome is good.

## Background

Congenital mesoblastic nephroma (CMN) is known as Boland's tumour or foetal renal hamartoma. It is a rare renal tumour that accounts for approximately 3% of renal tumours in children (1) and is the most common kidney tumour in the neonatal period (2). Histopathology of CMN can be divided into three types: classical, cellular, and mixed. Classical CMN is benign, similar to infantile leiomyoma, without a capsule. Classical CMN could infiltrate towards the renal parenchyma and have no apparent haemorrhage or necrosis. The prognosis of classical CMN is good. Cellular CMN is similar to infantile leiomyosarcoma, with high mitotic activity and potential invasive behaviour. It is a low-grade malignant tumour with apparent haemorrhage, degeneration and necrosis. Mixed CMN is a mixture of classical and cellular CMN, with the morphological characteristics of the two (3, 4).

Although CMN is the most common kidney tumour in the first month of life (54%–66%) (2, 5), only 11%–14% of cases can be detected by prenatal ultrasound (5, 6). Therefore, this article reported a case of CMN noticed in the third trimester and confirmed by postnatal histopathology. It is necessary to summarise the ultrasound features during the prenatal scan to provide better counselling to the future parent.

## Case presentation

**Figure 1:**
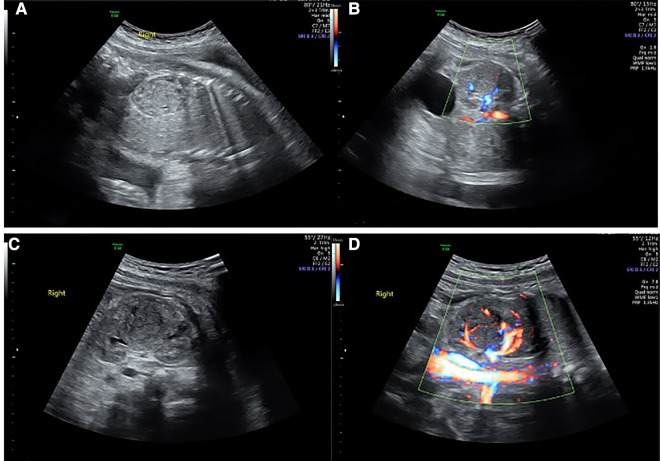
Renal solid mass at different gestational weeks. (**A,B**) showed the mass at 33 weeks and gestation by 2D and Colour Doppler. (**C,D**) showed the mass at 36 weeks of gestation by 2D and Colour Doppler.

**Figure 2:**
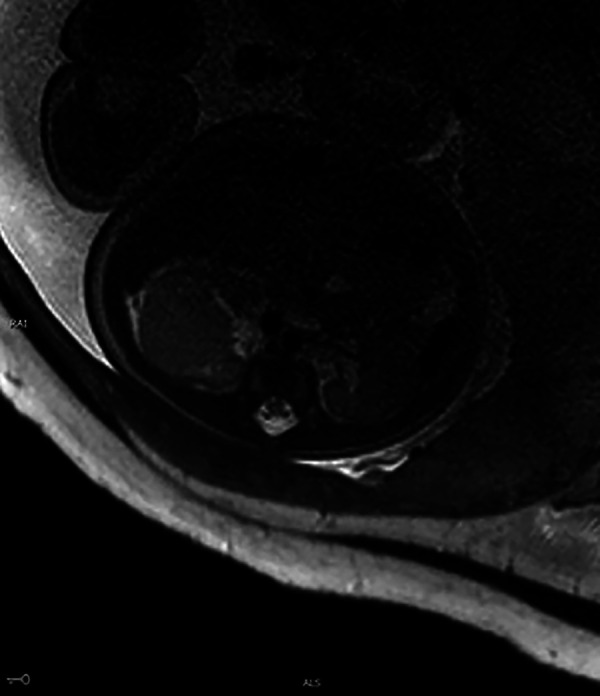
MRI of the foetus. A large, irregular mass (28 mm × 25 mm × 21 mm) was noted with isointense on T2WI.

A 31-year-old healthy Chinese woman with Gravida 3, parity 1, and abortion 1 received regular prenatal care at Peking University First Hospital. The first-trimester scan shows a crown-rump length (CRL) of 61.8 mm and a nuchal translucency (NT) of 1.77 mm. Maternal screening for Down's screening showed a low risk of trisomy 21 syndrome and trisomy 18 syndromes. An anomaly scan displayed no foetal structural abnormalities. The oral glucose tolerance test (OGTT) results were unremarkable. The pregnancy was uneventful until the growth scan at 29 weeks of gestation, considering susceptive large for gestation age (LGA) foetus. Hence, another OGTT was prescribed, and the result returned normal. During a referral ultrasound due to suspected LGA at 33 weeks of gestation, a medially echoic solid mass (27 mm × 28 mm × 22 mm) in the right kidney (Figures 1A,B) was detected. The echo of the mass was equivalent to that of the liver. Amniocentesis was recommended, but the patient refused. The following foetal magnetic resonance imaging (MRI) suggested a solid mass (28 mm × 25 mm × 21 mm) in the middle and upper right kidney with isointense on T1-weighted imaging (T1WI) and T2-weighted imaging (T2WI). The lesion had a well-defined boundary, and there were no apparent swollen lymph nodes in the surrounding area (Figure 2). However, the MRI did not cite a clear conclusion. After counselling with the future parents, they decided to continue the pregnancy and denied any family history of renal disease. The follow-up growth scan showed a growing solid mass with a measurement of 36 mm × 34 mm × 30 mm at 35 gestational weeks and a size of 41 mm × 33 mm × 31 mm at 36 gestational weeks (Figure 1C). Rich blood flow signals were around and inside the mass (“ring” sign, Figure 1D). The RI of the blood flow was 0.60 and 0.71, respectively. At 37 weeks of gestation, the pregnant woman underwent a caesarean section due to gestational hypertension and previous history of caesarean section (CS). The Apgar score at 1 min and 5 min were both 10. During the neonatal routine physical examination, the mass was palpable in the right hypochondriac region, with a diameter of approximately 3 cm.

**Figure 3:**
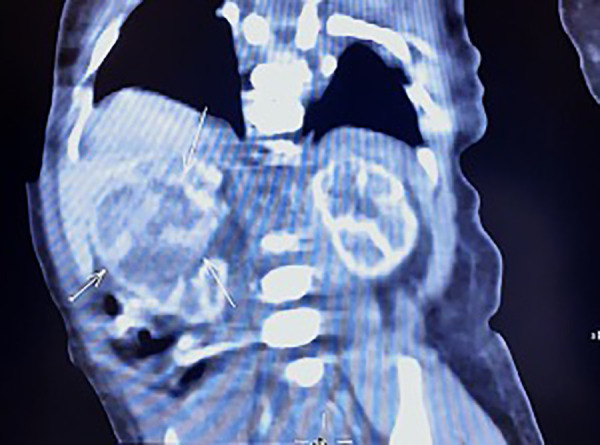
Enhance CT image of the newborn. A large, heterogeneous mass was noted in the middle of the right kidney with a size of 26 mm × 26 mm × 25 mm.

**Figure 4:**
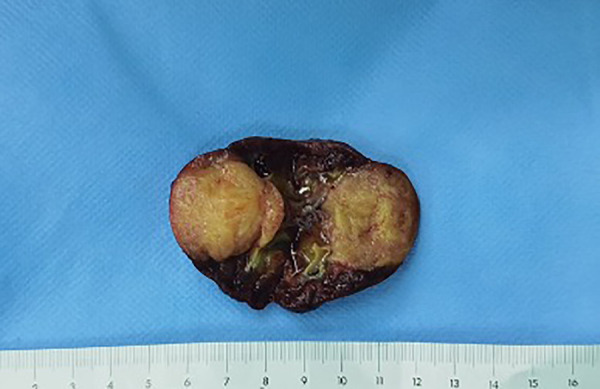
Right kidney was resected radically with a solid mass in the middle, yellow and white inside.

**Figure 5:**
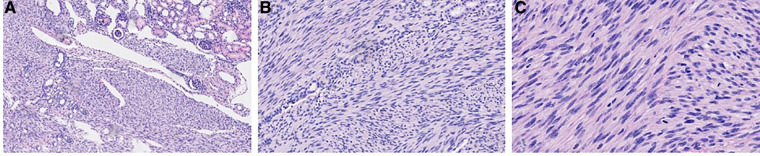
Microscopic images with haematoxylin and eosin (H&E) stain at different magnifications. (**A**) 100× magnification; (**B**) 200× magnification; (**C**) 400× magnification.

The neonate was transferred to the paediatric ward directly. An enhanced computerized tomography (CT) was performed on the 2nd day after birth, which suggested nephroblastoma with a measurement of 26 mm × 26 mm × 25 mm (Figure 3). The neonate underwent radical nephrectomy on the 9th day after birth with the whole affected kidney, the fatty tissues surrounding the kidney, and a portion of the tube connecting the kidney to the bladder (ureter) removed (Figure 4). The postoperative pathological report indicated an intrarenal nodular mass (31 mm × 27 mm × 20 mm), similar to prenatal imaging and postnatal CT measurements. The pathologist reported that the mass was formed by clusters of spindle cells (mild-moderate pleomorphism). It invaded the surrounding nephrons. The microscope findings demonstrated visible mitotic figures (20 cells/10 HPF), but no necrosis, nerve invasion or vascular tumour thrombi were noted (Figure 5). Molecular pathology suggested the absence of ETV6-NTRK3 fusion signals. Above all, the neonate was diagnosed with classical CMN and discharged on the 9th day after surgery.

The use of all data was approved by the Institutional Review Board of the Peking University First Hospital with the project number of 2013 [572]. Written informed consent was obtained from the patient for publication of this case report.

## Discussion

Bolande et al. (7) first reported CMN in 1967 and described its pathological tissue morphology in 1973 (8). However, CMN is the most common benign tumour in the first six months of life (2). However, most studies on foetal CMN are case reports (9–12). Lin et al. reported the first case of live birth diagnosed with foetal CMN in Taiwan. Patil et al. demonstrated a fetal CMN complicated with oligohydramnios and small for gestational age (SGA) foetus. In our case, the foetus manifested polyhydramnios and LGA. However, an intrauterine death (IUD) occurred in the case of CMN due to renal failure (11). In a study by Chen et al., the perinatal characteristics of 11 CMN patients were retrospectively studied. The patients with CMN detected by prenatal ultrasound had an average gestational age of 35 weeks (25–39 weeks) (13). In our case, the renal mass was first seen at 33 weeks of gestational age and grew larger gradually. The literature reported that CMN was more common in male infants (male: female ratio 1.5–2:1) (14, 15); however, the neonate was female in this report.

The prenatal ultrasound characteristic of CMN is mainly a lateral, single round or oval mass, with a solid or cystic-solid nature and a well-defined boundary with the surrounding organs. On colour doppler imaging, rich blood flow signals are observed inside and around the mass, described as a “ring” sign (16).

Polyhydramnios occurs in 15%–36.4% of CMN cases, which increases the risk of premature birth. The cause of polyhydramnios may be high blood perfusion in the kidney and compression of the surrounding intestine (13, 17). A foetus with CMN and oligohydramnios died one week after discovering the renal mass. Therefore, decreasing amniotic fluid volume could indicate renal failure, a predictor of poor prognosis for foetuses with CMN (11). Moreover, CMN in some foetuses could be complicated with other structural abnormalities, such as digestive system abnormalities, polydactyly, hydrocephalus, and Beckwith-Wiedemann syndrome (17). No other structural abnormalities were observed in this case, and the amniotic fluid did not increase throughout the gestational period.

In a Chinese report, three pregnant women terminated pregnancies due to an erroneous prenatal ultrasound diagnosis of foetal renal mass, while the autopsy turned out to be CMN (18). Therefore, improving the ability to diagnose and differential diagnosis is essential. Apart from CMN, the other foetal renal masses included Wilm's tumour (nephroblastoma) and neuroblastoma. Although Wilm's tumour is more common among children aged 3–4, the foetal type was reported occasionally. Rampersad et al. (19) and Vadeyar et al. (20) wrote that Wilms tumour in foetuses clinically manifests as polyhydramnios, foetal oedema, and foetal distress. The prenatal ultrasound of Wilm's tumour demonstrated a solid and cystic mass (21). However, Wilms tumour was difficult to distinguish from cellular CMN on prenatal ultrasound. Neuroblastomas originate from the adrenal gland. Therefore, it is necessary to determine whether the ipsilateral adrenal gland is present and assess the relationship between the mass and the adrenal gland. Neuroblastomas' boundaries are poorly defined and often contain haemorrhage, cystic degeneration and calcification. It is challenging to distinguish renal neuroblastomas from CMN when neuroblastoma is located in the kidney (22).

The correlation between foetal imaging findings and the histopathological types of CMN has been reported in the literature. Therefore, the ultrasound features of foetal CMN could correspond to a specific histopathological type. For example, a solid renal mass suggested classical CMN, while a renal mass with cystic degeneration, necrosis, and haemorrhage indicated cellular CMN (23). The ultrasound finding, in this case, was a well-defined solid mass, which was consistent with the final pathological result of classical CMN.

In a review by Gooskens et al., the classical, cellular, mixed, and classical/mixed CMN accounted for 39%, 42%, 10%, and 9%, respectively. Among the 251 cases of CMN, 208 (83%) were stage I/II, and only 42 (17%) were stage III (17). In CMN, the most common chromosomal gene mutations are trisomy 11 and t (12;15) (P13; q25) (24). The postoperative pathology for this case suggested classical CMN, and molecular pathology did not reveal ETV6-NTRK3 fusion signals.

The prognosis of infants with CMN is good, and the factors affecting the outcome include the pathological type and stage (25). Surgical treatment is required for CMN after birth. The primary surgical method is radical nephrectomy. Adjuvant chemotherapy suits patients with relapsed, high-stage (stage III or higher), or late-onset cellular CMN (14). The 5-year survival rate is 95%. In a study focused on renal tumours in infants within seven months of birth, the 5-year event-free survival (EFS) was 94%, and the 5-year overall survival was 96% (2). In another study reported by the German Society of Paediatric Oncology and Haematology (GPOH), the EFS was 100% in children with CMN diagnosed within three months of birth, and 83% in children with CMN diagnosed beyond three months after birth (6). Since CMN recurrence occurs in 4% of patients within one year, postoperative follow-ups should be performed at least one year after surgery (17).

Although CMN has been reported and discussed in previous studies, our case detailed all relevant imaging and postnatal management. Meanwhile, our case especially highlighted the contribution of prenatal ultrasound in the prenatal diagnosis of renal mass. More experience should be accumulated in MRI and CT of CMN.

## Conclusions

CMN could be detected prenatally, mainly during late pregnancy. Therefore, it is necessary to perform a detailed foetal ultrasound in the third trimester to detect structural anomalies, such as renal mass. Early diagnosis of foetal renal mass could favor the postnatal management and improve the neonatal prognosis.

## Data Availability

The datasets obtained and/or analyzed during the current study are available from the corresponding author on reasonable request.
